# Adenomyoma of the small intestine a rare pathological lead point for intussusception in an infant

**DOI:** 10.1186/2193-1801-3-616

**Published:** 2014-10-18

**Authors:** You-Jung Bak, Udo Rolle, Stefan Gfroerer, Henning C Fiegel

**Affiliations:** Department of Pediatric Surgery and Pediatric Urology, Johann Wolfgang Goethe University of Frankfurt, Theodor-Stern-Kai 7, D-60590 Frankfurt am Main, Germany

**Keywords:** Intussusception, Intestinal adenomyoma, Pathological lead point

## Abstract

**Introduction:**

Intussusception is a typical abdominal emergency in early childhood.

**Case description:**

We report a case of an infant in the typically affected age group with an intussusception triggered by a rare benign intramural intestinal adenomyoma as a pathological lead point. The infant had the typical symptoms of a recurrent idiopathic ileocolic intussusception.

**Discussion and evaluation:**

Idiopathic intussusception is frequent in the infant age group. Contrary to that, reports on pathological lead points for intussusceptions are sparse in the toddler age.

**Conclusions:**

That case illustrates that even in intussusceptions in the typically affected age group, it is important to be aware of pathological lead points, especially if the intussusceptions are recurrent.

## Background

Intussusception occurs when a proximal part of the bowel invaginates into a more distal part, typically within the ileocoecal region, which occurs commonly in infants and children between 3 months and 4 years of age. Typical symptoms in these patients include a triad of acute abdominal pain, vomiting and bloody stools; however, regularly, patients present with variable, non-specific symptoms. Ultrasonography is the established standard for diagnosis of intussusception and has a high sensitivity and specificity (Lehnert et al. 2009). Idiopathic intussusception occurs due to swollen mesenteric lymph nodes in patients in the typically affected age group that have been affected by viral infection or non-specific immunologic factors. If recurrent intussusception or intussusception occur in older children, the presence of a pathological lead point must be considered. Herein, we report and discuss the case of an infant in the typically affected age group with an ileocolic intussusception triggered by an adenomyoma of the distal ileum wall, a rare benign intramural intestinal tumor, acting as pathological lead point.

### Case description

**Figure 1:**
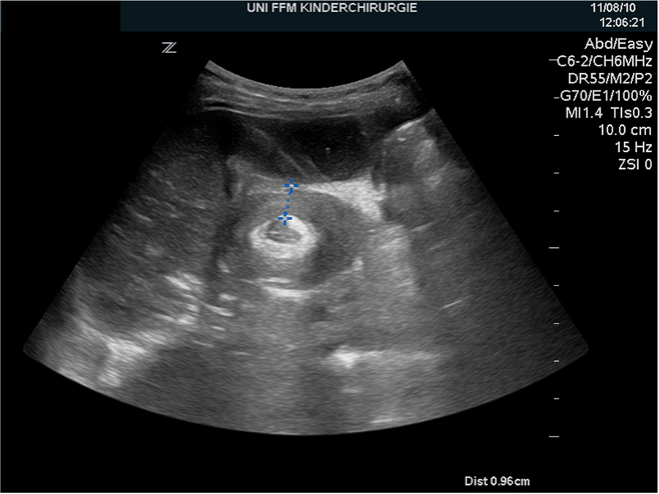
**Ultrasonography of the right upper abdominal quadrant revealed the typical ultrasonographic “target sign” of ileocolic intussusception after recurrent symptoms in the reported patient.**

**Figure 2:**
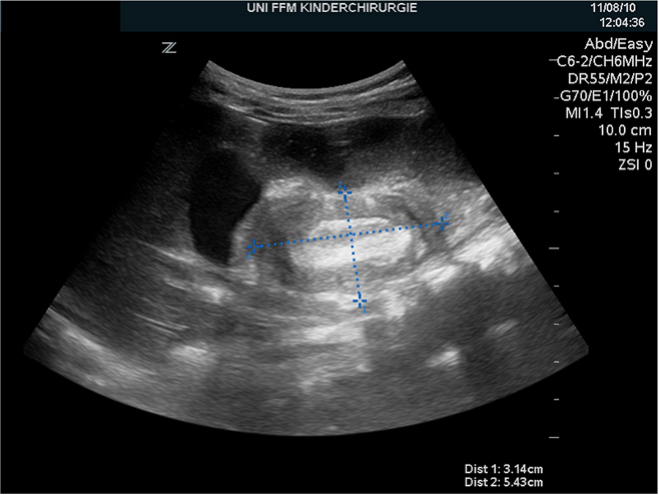
**Ultrasonography of the right upper abdominal quadrant revealed the typical ultrasonographic finding of “pseudo-kidney”.**

**Figure 3:**
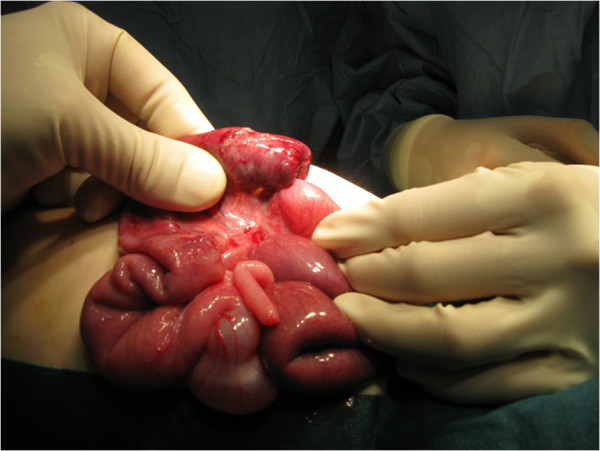
**Intraoperative findings of the ileocolic intussusception before manual reduction.**

**Figure 4:**
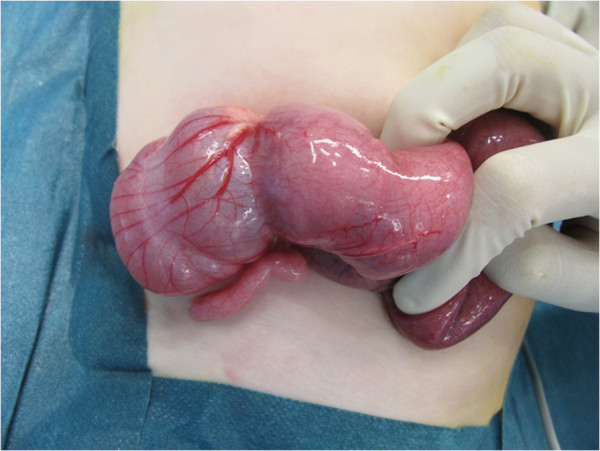
**Intraoperative findings of the palpable mass in the distal ileum after reduction of the intussusception.**

A previously healthy 11-month-old girl was admitted to our department with a 2-day history of colicky abdominal pain, intermittent agitation and sudden screaming. There were no episodes of bilious vomiting, bloody stools or fever. An ileocolic intussusception was diagnosed externally by ultrasonography, and immediate ultrasonography-guided hydrostatic reduction was attempted. Because complete reduction could not be achieved, the infant was transferred to our hospital. Physical examination showed a lethargic, dehydrated infant with a distended but nontender abdomen and decreased bowel sounds. Ultrasonography confirmed the ileocolic intussusception. Colonic enema reduction was performed immediately with successfully reposition, proved by ultrasound. The infant was rehydrated overnight, showed no symptoms the following morning and tolerated drinking well. Twelve hours after reduction, the infant presented again with crampy abdominal pain and vomiting. Ultrasonography showed again the typical findings of ileocolic intussusception (Figures 1 and 2). Repeated hydrostatic reduction was not successful. Therefore, emergency surgery was indicated. During laparotomy, an ileoileocolic intussusception was identified and reduced (Figure 3). After reduction, a palpable intraluminal mass presented as possible lead point of the intussusception approximately 10 cm from the ileocecal valve (Figure 4). Segmental resection of the ileum and reanastomosis were performed. The further recovery period was uneventful, and the infant was discharged 6 days after the operation.

**Figure 5:**
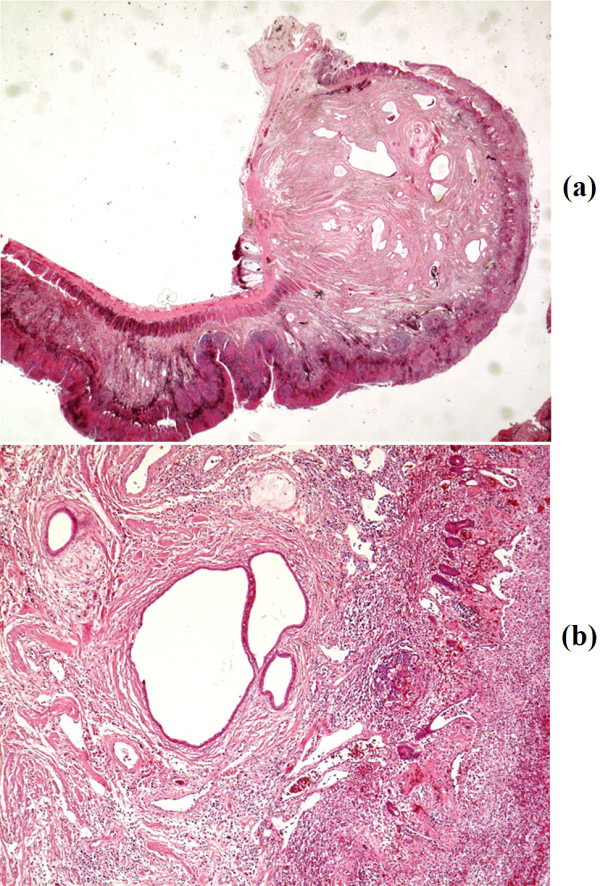
**Histological image: Adenomyoma localised in the submucosa of the ileum.** In addition a necrotising enteritis is shown. Primary magnification 0.5 **(a)** and 4× **(b)**.

Pathological findings were as follows. The mass was a 1×1×1 cm polypoid lesion covered with hemorrhagic and partly necrotic mucosa. Microscopically, the tumor was located in the submucosa and composed of glandular structures lined by mucin-secreting columnar epithelium and smooth muscle bundles (Figure 5). These findings were compatible with the diagnosis of adenomyoma of the ileum. Elsewhere, the ileum showed severe mucosal ulceration and necrosis in addition to subtotal perforating enteritis with hemorrhagic infarction, all of which were consistent with changes resulting from the intussusception.

### Discussion and evaluation

Intussusception is a common cause of bowel obstruction in infants and toddlers, with the greatest incidence in infants aged 3–9 months (Lehnert et al. 2009, Gfrorer et al. 2009). There is a seasonal incidence, with peaks in spring and autumn resembling the most typical periods of seasonal gastroenteritis and respiratory tract infections. Most infants do not have a specific lead point. Hypertrophied Peyer’s patches and reactive lymph node hyperplasia, which result from prior viral infection, can serve as a lead point for idiopathic intussusception. Specific lead points (e.g., Meckel diverticulum, intestinal polyps, lymphomas, and intestinal duplication) are more commonly found in older children and adults. Ultrasonography is the preferred diagnostic tool in intussusception and has a sensitivity of 98-100% and a specificity of 88-100% (Lehnert et al. 2009, Gfrorer et al. 2009). Hydrostatic reduction under ultrasound control and contrast enema are established therapies for the treatment of intussusception, with a success rate of 70-90% (Lehnert et al. 2009, Gfrorer et al. 2009). Immediate surgery is indicated in patients who have peritonitis, sepsis, evidence of perforation, unsuccessful non-operative repositioning or a clear finding of pathological lead points. In cases occurring in individuals not in the typical age group or in cases of recurrent intussusceptions, a pathological lead point must be excluded.

Adenomyoma of the gastrointestinal tract is a rare benign lesion localized at the stomach, small intestine and biliary ducts (Zhu et al. 2010). Adenomyoma of the stomach is usually asymptomatic. Its occurrence in the small intestine of children is extremely rare. However, in the small intestine, intussusception is its most common complication, which has been reported in 13 cases so far (Table 1). The reported cases had significantly varied ages, with a range from 2 days to 82 years. In our case, the infant was of the typical age and had the symptoms most commonly associated with idiopathic ileocolic intussusception, but the intussusception was nonetheless due to a pathological finding.

**Table 1:** **Previous reported cases of adenomyoma in intussusception**

No.	First author	Year	Age	Surgical diagnosis	Histopathology
1	Schwartz et al.	1958	8 months	intussusception	myoepithelial hamartoms
2	Gal et al.	1986	82 years	intussusception	adenomatous hamartoma
3	Kim et al.	1990	7 years	intussusception	adenomyoma
4	Gal et al.	1991	9 months	intussusception	adenomyoma
5	Lamki et al.	1993	1 year	intussusception	adenomyoma
6	Serour et al.	1994	3 years	intussusception	adenomyoma
7	Chan et al.	1994	5 months	intussusception	adenomyoma
8	Gonzalvez et al.	1995	2 years	intussusception	adenomyoma
9	Yamagami et al.	1997	4 months	intussusception	adenomyoma
10	Lee et al.	2001	18 years	intussusception	adenomyoma
11	Park et al.	2003	7 months	intussusception	adenomyoma
12	Mouravas et al.	2003	18 months	intussusception	adenomyoma
13	Takeda et al.	2011	68 years	intussusception	adenomyoma
	here described case	2013	11 months	intussusception	adenomyoma

## Conclusions

Adenomyoma of the small bowel is a rare cause of intussusception in all age groups. The here presented case shows, that even in patients where intussusceptions occur in the typically affected age group, it is important to be aware of pathological lead points, especially in recurrent intussusceptions.

### Consent

Written informed consent was obtained from the parents for the publication of this report and any accompanying images.
